# Evaluation of the Mycorrhizal Potential of Date Palm (*Phoenix dactylifera* L.) Rhizosphere Soils in the Figuig Oasis (Southeastern Morocco)

**DOI:** 10.3390/jof9090931

**Published:** 2023-09-15

**Authors:** Elmostafa Gagou, Khadija Chakroune, Mahmoud Abbas, Touria Lamkami, Abdelkader Hakkou

**Affiliations:** 1 Laboratory of Bioresources, Biotechnology, Ethnopharmacology and Health, Faculty of Sciences, University Mohammed First, BV Mohammed VI BP 717, Oujda 60000, Morocco; khadija.chakroune@ump.ac.ma (K.C.); kadahakkou@yahoo.fr (A.H.); 2 Administrative Centre, Laboratory of Water Analysis of Figuig (LAEF), Municipality of Figuig, BP 121, Figuig 61000, Morocco; mabbasaacf@hotmail.com; 3 Department of Research in Drug Development, Faculty of Pharmacy, Université Libre de Bruxelles, Bvd du Triomphe, 1050 Brussels, Belgium; touria.lamkami@ulb.be

**Keywords:** arbuscular mycorrhizal fungi (AMF), Figuig oasis, mycorrhizal soil potential, rhizosphere

## Abstract

Date palm, an important crop in Morocco and many other arid regions around the world, faces significant challenges from wind, water shortages, and salinization, which contribute to vegetation loss and soil degradation in the harsh environmental conditions of oasis ecosystems with low soil fertility. Protecting and regenerating these degraded lands is crucial for sustainable agriculture and improving the dryland ecosystem. Arbuscular mycorrhizal fungi (AMF) comprise a vital element in this dynamic within the microflora of the soil rhizosphere. This study evaluated the potential in mycorrhizal soil and identified AMF in date palm rhizospheres in eight locations within the Figuig oasis (southeastern Morocco). This study found that Extension and Zenaga had more mycorrhizal propagules than other locations. Replanted maize (*Zea mays* L.) in these soils exhibited higher mycorrhization rates (91–93%) compared to that in other locations, with the Lamaiz site registering the lowest rate (39%). The phosphorus content was negatively correlated with the AMF spore frequency, intensity, and density, while a positive correlation was detected between the soil pH and the AMF spore frequency and density. The morphological identification of spores revealed *Glomus* as the predominant species, along with *Acaulospora* and *Sclerocystis*. This study represents an initial step toward the potential application of these fungi in environmental conservation and sustainable agriculture in arid regions.

## 1. Introduction

The date palm (*Phoenix dactylifera* L.) is known as a vital fruit crop on a global scale, offering unique environmental and nutritional advantages. This tree species holds immense promise in combating global warming by effectively sequestering carbon dioxide, surpassing the capabilities of many other trees [1]. Moreover, the date fruit boasts a rich array of essential nutrients, making it an ideal food choice, with considerable potential health benefits [2,3]. In the Middle East and in North Africa, the date palm assumes a pivotal role, not only economically and ecologically, but also socially, serving as a crucial source of income for oasis farmers [4]. Furthermore, date palm cultivation fosters a favorable microclimate, making agriculture feasible even under harsh desert conditions [5]. Despite their significance, date palm oases face various challenges that endanger their sustainability. Climate change has imposed cumulative effects, and soil salinization, caused by factors including water resource limitations, the increased salinity of irrigation water, and a reduced drainage water discharge in agricultural lands, emerges as a pressing threat to these oases [6,7,8,9,10]. A particular case illustrating these challenges is the Figuig oasis in the southeastern part of Morocco, which suffers from significant threats to its efficiency and biodiversity. The scarcity of water resources due to climate change, along with the soil salinity and the presence of *Fusarium* wilt (Bayoud disease), caused by *Fusarium oxysporum* f. sp. *Albedinis*, exacerbates the situation [11,12,13,14].

Faced with these challenges, and with the aim of mitigating the adverse effects of biotic and abiotic stress without resorting to costly modern techniques, it is essential to focus research on alternative approaches such as the use of below-ground microorganisms that are likely to promote the growth of date palms in these fragile environments. Among these microorganisms, arbuscular mycorrhizal fungi (AMF), which live in symbiosis with the majority of agricultural and horticultural important crops, are of great interest. These fungi provide numerous benefits for plant growth and health in stressful environments [15,16]. In arid conditions, for example, AMF-colonized plants have demonstrated a greater drought tolerance [17] and better access to phosphorus than noncolonized plants [18]. AMF can also improve the stability of soil aggregates [19], which is crucial in erosion-prone sandy soils. In extreme desert ecosystems, AMF play a key role in vegetation development. For example, inoculation with AMF has been shown to enhance water and nutrient uptake among desert succulents [20].

Research by Meddich et al. [21] revealed the important role of native AMF isolated from the Aoufous palm grove in the tolerance of date palms to water deficit and *Fusarium* head blight. These AMF also act as bioindicators, as the characteristics of agricultural soils can be determined on the basis of their mycorrhizal fungal communities. Despite their interest and importance, AMF are rarely used on farms, partly due to the incompatibility of the introduced isolate with local soil characteristics [22], leading to the disappearance of the introduced inoculant. It would, therefore, be wise to select indigenous isolates, adapted to the constraints of the Moroccan oasis environment. Indeed, the adaptation of AMF inoculants to specific environmental conditions has been widely documented [23,24,25]. It has been shown that AMF perform best when experimental conditions most closely resemble those of their native environment [25]. Therefore, it can be assumed that AMF isolated from desert ecosystems are better adapted to cope with the prevailing stress conditions and may exhibit unique physiological capabilities.

In this context, our work aimed to study the soils of the Figuig palm grove and assess their mycorrhizal status, with a view to their potential valorization. It is essential to characterize AMF isolates adapted to date palm ecosystems, as a prerequisite to future fundamental and applied research projects.

## 2. Materials and Methods

### 2.1. Study Area and Sampling

**Figure 1:**
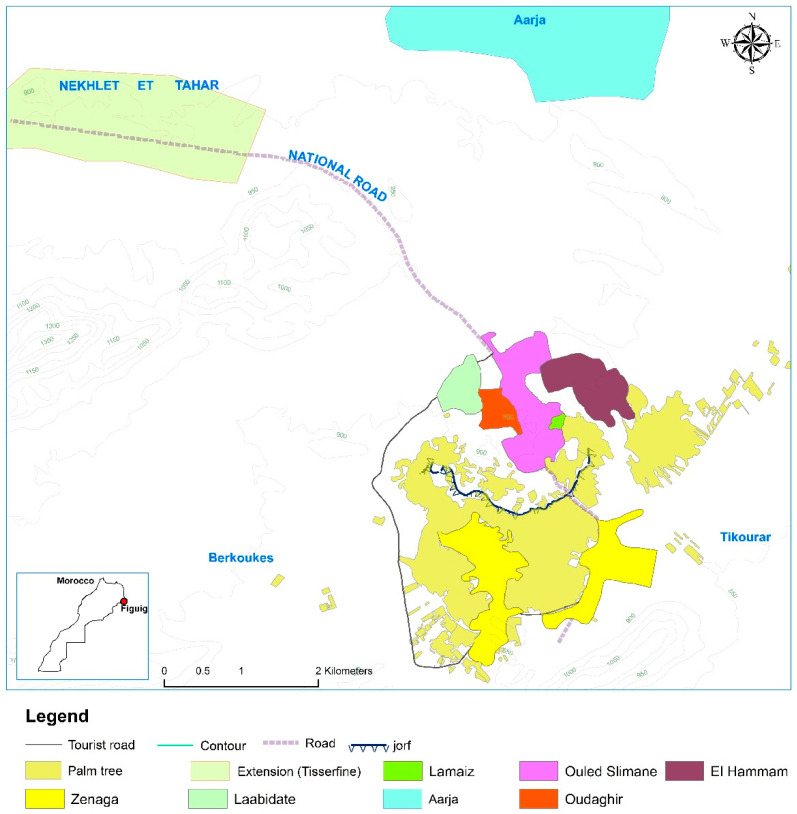
The region’s topography and the explored locales. A satellite view of Figuig oasis, displaying the sample collection sites.

The oasis of Figuig is subdivided into the following ksour: Zenaga (ZG), Oudaghir (OD), Lamaiz (LZ), Ouled Slimane (OS), Laabidate (LB), and Elhammam (EH), which were originally separate settlements but have since amalgamated, due to urban expansion. Two distinct sections, separated by the escarpment known as jorf, make up the region’s topography. The biggest ksar of Zenaga is situated at the lower level, and the remaining five ksour, and the associated palm groves, are located at the higher level, which rises to an altitude of 899 m above sea level. Extensive palm groves, also known as Extension (EX) and Aarja (AR), are located outside the city limits (Figure 1). With a careful selection of eight different sites, to ensure a full representation of the palm grove, each location was chosen in accordance with the corresponding palm grove.

In March 2019, we collected soil samples in each site, we randomly selected three replicated plots per site, each covering an area of 700 m^2^. Within each plot, we carefully chose seven date palm trees and collected four individual soil subsamples from each tree, at a depth of 0–30 cm and a 50 cm distance from the trunk. These subsamples were combined to create one composite sample for each plot, yielding a total of 24 composite samples for further analysis. 

### 2.2. Soil Physicochemical Analyses

The soil pH was measured with the use of a Biobase China pH-920 electrical pH meter equipped with a glass electrode, immersed in a mixture of distilled water and soil suspension (1:1), following the method outlined by Eaton et al. (2005) [26]. For the determination of the available phosphorus (Olsen P) in the soil samples, the extraction was conducted using 0.5 M NaHCO_3_ at pH 8.5, following the method of Olsen et al. (1954) [27]. Through the Walkley and Black method, the soil’s organic matter content was measured, as described by Mathieu and Pieltain (2003) [28]. Potassium (K) was extracted using a 1 M ammonium acetate solution, according to the protocol established by Mathieu and Pieltain (2003) [28]. The salinity was determined via the measurement of the EC (electric conductivity) of a 1:5 soil-to-water extract, according to He et al. (2012) [29]. The carbonate content (CaCO_3_) was measured via the volumetric method, with a Bernard calcimeter, as per the French Standard NF P 94-048 [30]. According to Ritchey et al. (2015) [31], the “Feel Method” was used to determine the soil texture.

### 2.3. Evaluation of the Number of Infective Propagules of AMF of the Investigated Soils

The evaluation of the number of infective AMF propagules of the different soils was based on the most probable number (MPN) method. This bioassay measures the presence or absence of AMF propagules (by observing colonization of the roots) in a dilution series of the soil, with the results interpreted as a probability estimate of propagule numbers from a statistical table [32]. The testing procedure began with the air-drying of the soil samples to remove moisture. The samples were then sieved to a particle size homogeneity of 2 mm. This sieving process prepared the soil for subsequent manipulation. After being sieved, the soil samples were diluted with sand that had previously been sterilized. This sterilization was achieved via the heating of the sand at 180 °C for three hours. Sterilization was essential for preventing contamination or the development of microorganisms that could have skewed the test results. Each soil sample was subjected to six distinct dilutions, with factors of 1/4, 1/16, 1/64, 1/256, and 1/1024. Each dilution was repeated five times, to yield statistically significant data and minimize the experimental error.

Next, plastic pots with a 200 mL capacity were used to hold the diluted soil samples. Each pot was filled with 100 g of diluted soil, which represented an exact quantity of nonsterile soil from each dilution. As a host plant, the maize plant (*Zea mays* L.) was purposefully selected as the symbiotic partner, due to its notable mycorrhizal dependency, considerable germination rate, early receptivity to mycorrhizal colonization, and prolific root production [33]. To achieve this, the maize seeds were subjected to a surface sterilization procedure, which entailed their immersion in a 10% *v*/*v* sodium hypochlorite solution for 10 min. Afterward, the seeds underwent a thorough rinsing procedure with sterilized water to ensure effective sterilization. After one week, each seedling was transplanted into the pot and carefully placed in the greenhouse, with precise control measures in place to maintain a constant temperature of 25 °C and 80% humidity. After one month of cultivation, the plants were extracted from their pots, and their root systems were cleansed and stained, according to the method described by Philips and Hayman [34]. This staining renders the AMF structures in roots visible. The roots were then cut into 1 cm segments and placed between a microscope slide and a cover slip for microscopic examination. A root system is said to be colonized by AMF if it contains at least one infection point, thus indicating the penetration of hyphae into the root. Using the following formula, the most probable number of propagules was determined:

Log MPN (Most Probable Number) = (x log a) − K

x represents the mean number of AMF-colonized plants. a represents the dilution factor. K values are available in the tables published by Fisher et al. (1949) [35].

### 2.4. Evaluation of AMF Spore Numbers and Identification of AMF Species

In parallel to the MPN method, direct extraction of spores from the soils was performed with the wet sieving method described by Gerdemann et al. (1963) [36]. This method required a series of sieves with progressively smaller sizes (500 µm, 250 µm, 100 µm, and 40 µm). The material retained by the final three sieves was collected in 50 mL Falcon tubes and centrifuged for 2 min at 900× *g* in the presence of a 70% sucrose solution. The supernatant solution collected from the 40 µm sieve was thoroughly rinsed with tap water. The extracted spores were then distributed in Petri dishes. To determine the quantity of spores in each area, five soil samples were examined under a stereomicroscope. The number of spores for each gram of soil was used to define the spore numbers that were found. In addition, the isolated spores were used to identify the AMF species. The morphological attributes of the spores, including characteristics such as the color, size, form, wall number, abundance, suspension hyphae, and internal structure, were assessed using a stereomicroscope. Permanent specimens created with a solution of polyvinyl alcohol, lactic acid, and glycerol (PVLG), as described by Koske et al. (1983) [37], and a mixture of PVLG and Melzer’s reagent, as described by Brundrett et al. [38], were used to test these characteristics. The morphological characterizations provided by AMF Phylogeny http://www.amf-phylogeny.com (accessed on 17 May 2023) and the International Culture Collection of Vesicular Arbuscular Mycorrhizal Fungi https://invam.ku.edu/species-descriptions (accessed on 17 May 2023) were used to identify the spores.

### 2.5. Frequency of Mycorrhization and Intensity of Maize Root Colonization

According to the method by Koske and Gemma [39], field-collected maize roots were evaluated for AMF root colonization. Briefly, the roots were cleaned in a 10% potassium hydroxide (KOH) solution at 90 °C for 10 min before being stained at 70 °C for 30 min with 2% Parker’s blue ink in 1% HCl containing 2% ink (Parker’s blue ink, manufactured by Parker Inc., New York City, NY, USA). The rate of colonization by AMF was calculated via the arrangement of 15 stained, 1 cm long roots on glass slides, and the calculation of the percentage of colonization. The availability of AMF structures (hyphae, arbuscules, or vesicles/spores) was determined via the examination of the hyphae, arbuscules, and vesicles/spores, and in 90 root fragments per location. According to Trouvelot et al. [40], the mycorrhizal colonization of the root system was scored on a basis of intensity (0 to 5). A value of 0 suggested the absence of AMF colonization (0%), whereas a score of 1 suggested the presence of minimal AMF structures (1%). The scores 2, 3, 4, and 5 indicated progressively higher levels of AMF colonization: 2 (1–10%), 3 (10–50%), 4 (50–90%), and 5 (above 90%), respectively. Mycocalc https://www2.dijon.inrae.fr/mychintec/Mycocalc-prg/download.html (accessed on 12 April 2023) was used to calculate the intensity (M%) and frequency (F%) of AMF colonization in the root system. F% = (total count of mycorrhizal root fragments/root fragments observed) × 100, whereas M% = (95n5 + 70n4 + 30n3 + 5n2 + n1/total root fragments observed) × 100, where n5, n4, n3, n2, and n1 represent the total numbers of fragments classed as 5, 4, 3, 2, and 1, respectively.

### 2.6. Statistical Analyses

The intensity and frequency of the mycorrhizal colonization, as well as the AMF spore numbers, were analyzed using one-way evaluations of variance (ANOVA 1), accompanied by Tukey’s test at a level of significance of 0.05. The Q–Q plot was utilized to examine the normality of the residuals. The correlation between the parameters of the mycorrhizal symbiosis and the chemical analysis of the soil was analyzed via Pearson correlation. SPSS statistical software (Version 21.0.0.0 Edition 32 bits) was utilized for the analysis (IBM SPSS Inc., Chicago, IL, USA.).

## 3. Results

### 3.1. Physical and Chemical Soil Properties

The physicochemical properties of rhizosphere soil were carefully examined to determine their influence on the distribution and abundance of AMF. The outcomes concerning the soil texture were determined with the utilization of the “Feel Method”. The data aggregation in Table 1 reveals the existence of two distinct soil textures: the sandy clay loam found at the AR and EX sites, and the clay loam prevalent at the other sites.

The measured soil properties, encompassing both physical and chemical aspects, displayed slight variations. The pH ranged slightly within the alkaline side, from 7.4 to 8.1. The soil exhibited a predominant calcareous nature, with the calcium carbonate (CaCO_3_) levels ranging from 8% to 45%. Similarly, the analysis of the organic matter content revealed higher percentages at the LZ and LB sites, reaching 1.8% and 1.7%, respectively. The ZG, OD, and EX sites recorded the lowest organic matter values, at 0.4% (Table 1). Likewise, the levels of phosphorus (P_2_O_5_) and potassium (K_2_O) exhibited variations between sites. The highest phosphorus concentration was found at the LZ site, while the OD site showed the highest potassium value. Conversely, the lowest phosphorus and potassium levels were observed at the ZG site. Regarding the salinity, all samples showed moderate variations, ranging from 0.1 to 1.2 (g/kg).

### 3.2. Number of Infective Propagules of AMF in the Soils

After one month of cultivation of the maize plants on the serial dilutions, the numbers of AMF-colonized plants on the different dilutions of the eight sites are as shown in Table 2. Remarkably, all the soil samples from the different sites exhibited a 100% mycorrhizal potential up to a dilution ratio of 1/64. However, at all the tested concentrations, only two sites, ZG and EX, showed this level of potential for mycorrhization. When maize was cultivated at moderate to high dilutions (1/256 and 1/1024), the AMF infection rates ranged from 40% to 80% in the soil samples from the OD, LZ, EH, LB, and AR sites.

### 3.3. AMF Root Colonization and Spore Density

**Figure 2:**
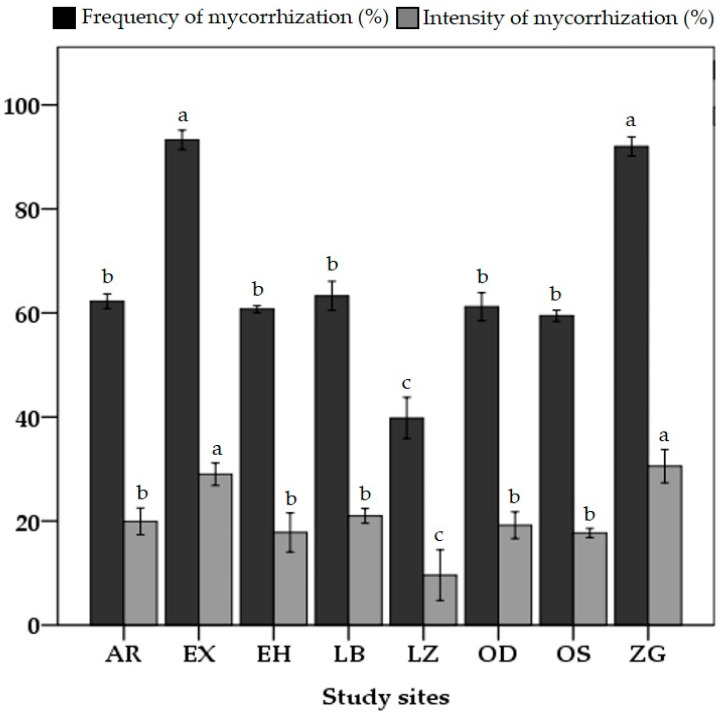
The frequency and intensity of mycorrhizal colonization in *Zea mays* roots from the eight study locations. Aarja (AR); Extension (EX); Elhammam (EH); Laabidate (LB); Lamaiz (LZ); Oudaghir (OD); Ouled slimane (OS); and Zenaga (ZG). The letters just above the bars reflect the statistically significant differences, as determined via the Tukey test (*p* < 0.05).

**Figure 3:**
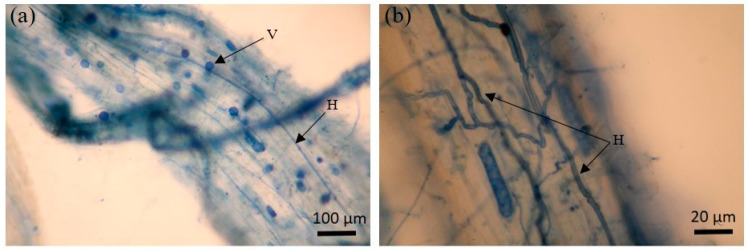

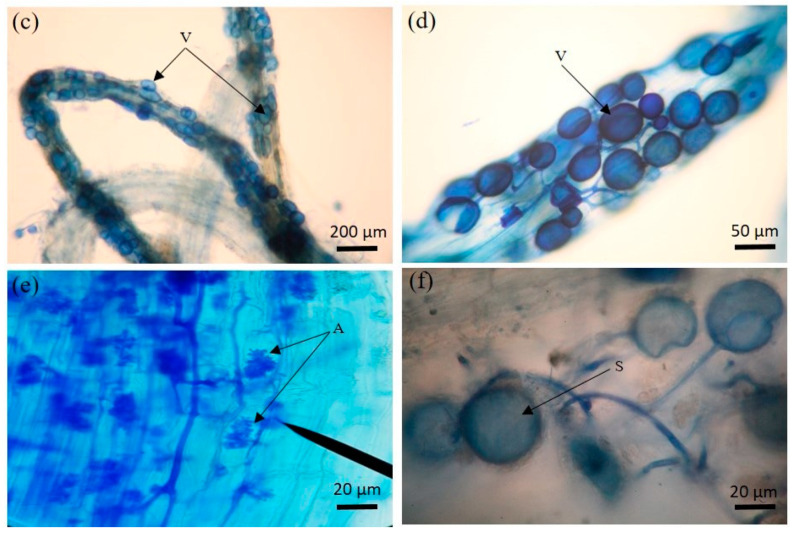
AMF structural colonization in *Zea mays* trap plants. Hyphaes and vesicles colonization (**a**); hyphae colonization (**b**); vesicles colonization (**c**,**d**); arbuscules colonization (**e**); spores colonization (**f**). Notes: vesicle (V); hypha (H); arbuscule (A); intraradical spore (S).

**Figure 4:**
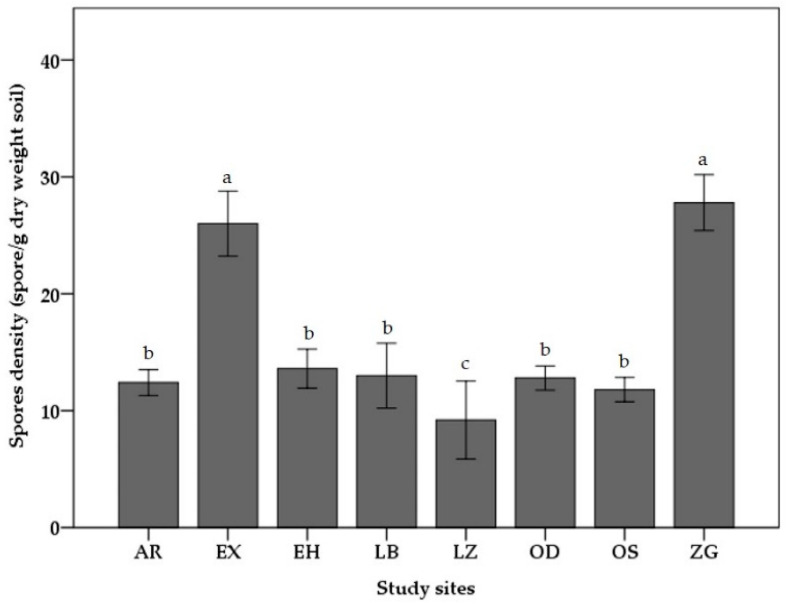
Density of spores in rhizosphere soil samples from eight study sites. Aarja (AR); Extension (EX); Elhammam (EH); Laabidate (LB); Lamaiz (LZ); Oudaghir (OD); Ouled slimane (OS); and Zenaga (ZG). The letters just above the bars indicate significant variations, according to the Tukey test (*p* < 0.05).

The frequency and intensity of mycorrhization in *Zea mays* roots reveal significant differences between the sites after one month of cultivation (*p* < 0.05) (Figure 2 and Figure 3). The ZG and EX sites are distinguished through their high frequency of mycorrhization, which reaches 91% and 93%, respectively. In terms of the colonization intensity, these two locations exhibit a similar pattern. In contrast, the LZ site demonstrates the lowest value, with its frequency and intensity percentages failing to exceed 39% and 9%, respectively. The frequencies vary between 50 and 60%, and the intensity levels vary between 10 and 20% at the remaining sites, which are not significantly different (*p* > 0.05). In terms of the spore density, the soils of the ZG and EX sites differ by a considerable number of mycorrhizial propagules that extend from 25 to 28 propagules per 1 g of soil and are significantly different among the remaining sites (*p* < 0.05) (Figure 4). In contrast, only nine propagules per gram of soil were recorded at the LZ site. Other sites contained 11 to 13 propagules per gram of soil, a density that was relatively comparable.

### 3.4. Correlations among AMF Parameters and Soil Chemical Characteristics

**Figure 5:**
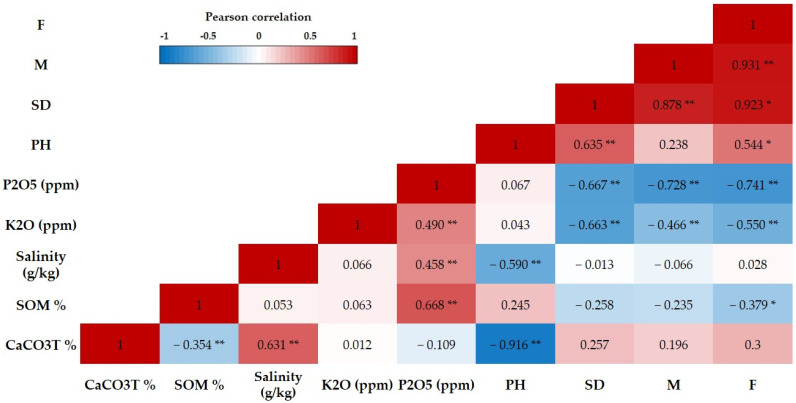
Heat map of Pearson’s correlation coefficient between the Phoenix dactylifera L. rhizosphere soil chemical characteristics and AMF parameters. Frequency of mycorrhization (F); intensity of mycorrhization (M); spore density (SD); (*) the significance of the correlation is at the 0.05 level; (**) the significance of the correlation is at the 0.01 level.

The Pearson’s coefficient (r) was employed as a metric for the prompt detection of correlations among variables. The value of correlation ranges from −1 to 1, with −1 representing a negative correlation, 1 representing a positive correlation, and 0 indicating the absence of a linear correlation. The heat map in Figure 5 displays the correlation coefficients between various AMF parameters. These parameters include the frequency and intensity of mycorrhizal colonization, spore density, and soil chemical characteristics in the date palm rhizosphere. Correlation analyses revealed significant associations between these variables. Specifically, the mycorrhizal colonization frequency correlated positively with intensity of mycorrhization and spore density, showing correlation coefficients (r) of +0.931 and +0.923, respectively. In addition, the mycorrhizal frequency was negatively correlated with the soil phosphorus (*p* = 0.005), potassium (*p* = 0.03), and organic matter (*p* = 0.04) levels. The results also indicate that the soil pH was positively associated with the mycorrhizal frequency (*p* = 0.02) and spore density (*p* = 0.007). On the other hand, the spore density was negatively correlated with the phosphorus (*p* = 0.006) and potassium (*p* = 0.007) levels, while a positive correlation was observed with the soil pH (*p* = 0.006). Finally, the intensity of mycorrhization showed a negative correlation with the phosphorus (*p* = 0.007) and potassium (*p* = 0.008) levels.

### 3.5. Morphological Identification of AMF

**Figure 6:**
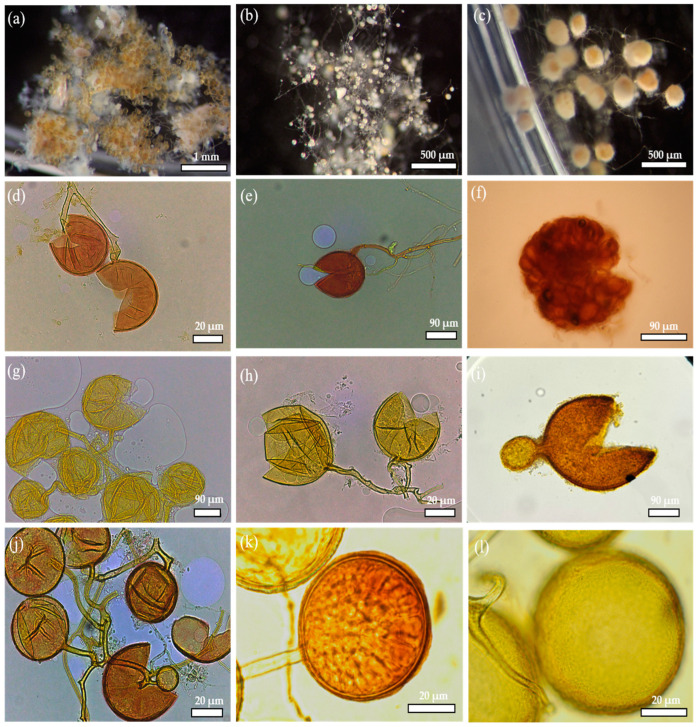
Numerous AMF spores were found in the rhizosphere of the Figuig oasis. *Glomus* sp. from pot cultures (**a**–**c**); *Glomus* sp. mounted in PVLG/Melzer (**d**,**e**); *Sclerocystis* sp. mounted in PVLG/Melzer (**f**); *Glomus* sp. mounted in PVLG (**g**,**h**); *Acaulospora* sp. mounted in PVLG (**i**); *Glomus* sp. mounted in PVLG (**j**,**k**); *Scutellospora* sp. mounted in PVLG (**l**).

The examination of the soil samples taken from the rhizosphere of the sanctuary of the Figuig oasis involved isolating and identifying mycorrhizal spores. The identification process revealed the presence of 11 species, representing five genera: *Rhizophagus*, *Funnelifomis*, *Sclerocystis*, *Scutellospora*, and *Acaulospora* (see Table 3 and Figure 6). The genus *Rhizophagus* was the most prevalent, with a response rate between 70 and 90%, and a higher spore density, characterized by a generally multilayered wall that blended with the wall of the subtending hyphae (Figure 6d,e). The proportions of the genera *Scutellospora* and *Acaulospora* ranged between 10 and 20%. *Acaulospora* is distinguished by spores that separate from a sporiferous saccule and then become sessile (Figure 6i). There was only one species represented within this genus, *Acaulospora* sp., whereas *Sclerocystis* was fairly rare. In addition, similar proportions of *Funneliformis* sp and *Rhizophagus* sp were isolated from all soil samples.

## 4. Discussion

The present study focused on the evaluation of the mycorrhizal soil potential and the identification of AMF in the rhizosphere of date palms in the Figuig oasis. The mycorrhizal status must be studied to exploit all the beneficial properties that the symbiotic relationship between mycorrhizal fungi and plants can provide. When confronted with abiotic stress, plants develop a variety of strategies to avoid it or increase their tolerance. Many research studies have discovered specific strategies that minimize the negative impact of stress on plant growth [41,42]. According to studies published by Oyediran et al. (2018) [43], plants in arid regions have the capacity to produce large quantities of sugars and amino acids in order to withstand environmental stresses. Moreover, the low phosphorus content of arid soils promotes symbiotic interactions between plants and fungi, which may increase the diversity in mycorrhizal fungal spores in these regions.

An analysis of the correlation between the chemical parameters of the soil under study and the parameters of mycorrhizal symbiosis (Figure 5) supported this. Marschner and Cakmak (1986) [44] observed that the presence of certain chemicals at high concentrations tended to cause a frequent reduction in the rate of mycorrhization. Amijee et al. (1989) [45] also confirmed this trend with regard to phosphorus. In fact, root colonization by mycorrhizal structures is highest in environments where the phosphorus concentration is kept low, but decreases as the phosphorus concentration increases. Even very low phosphorus concentrations have been associated with a decline in mycorrhization rates [46]. As an example, our study site LZ has the highest phosphorus content (100 ppm), but the frequency and intensity of mycorrhization remain very limited, at around 39% and 9%, respectively. On the other hand, at the ZG site, where the phosphorus levels reach 20.3 ppm, the frequency and intensity of mycorrhization are 91.99% and 30.56%, respectively. In addition, similar observations have also been made regarding reduced levels of potassium and organic matter. These findings are consistent with those of Oehl et al. (2010) [47]. Furthermore, Bhat et al. (2014) [48] demonstrated that soil potassium and phosphorus availability and AMF root colonization can have a significant relationship.

A significantly positive correlation was found between the colonization frequency and spore density in the soil samples and the pH in our study sites, which ranged from 7.4 to 8.1. Toh et al. (2018) [49] found that the soil pH in the rhizosphere had significant effects in terms of the number of spores present in the soil and the rate of root colonization by AMF. Our results are in accordance with these findings. Numerous investigations have provided additional evidence for the correlation between mycorrhizae and soil pH variations. Converging trends indicate a positive correlation between mycorrhizal density and the pH, indicating that the infection rate increases as the pH rises [50,51]. In addition, Bainard et al. (2014) [52] reported that certain species had a distinct preference for environments characterized by acidic soil. Variations in the soil pH had a significant impact on the diversity of arbuscular mycorrhiza populations [53], a finding that was equally important. In contrast, research by Bainard et al. (2014) [52] produced a contrasting finding, showing no significant relationship with the soil pH. The variability in the optimal pH values, depending on the various mycorrhiza species present, helped to explain this lack of a significant correlation. Indeed, the influence of the pH can vary from one species to another. A study by Melo et al. (2017) [54] also highlighted this diversity, indicating that members of the Acaulosporaceae family showed a negative correlation with the pH, while members of the indeterminate Glomoid group showed a positive correlation with pH fluctuations. Other soil parameters, such as the salinity and total limestone content, exhibited no significant correlation. These findings are consistent with those of Oehl et al. [47].

A variety of AMF spore genera were isolated and characterized from the soil samples, as shown in Table 3 and Figure 6. The characterization of spores focused mainly on characteristics such as the spore form and color, the number of wall layers, and any other structures associated with AM fungi. Figure 6 shows that the mycorrhizal fungi isolated from the soil belonged to the genera *Glomus* sp., *Acaulospora* sp., *Funneliformis* sp., *Rhizophagus* sp., *Sclerocystis* sp., and *Scutellospora* sp. These species are typically found in arid and semiarid habitats. Chebaane et al. (2020) [55] reported the presence of several different isolates, including *Funneliformis* sp. and *Rhizoglomus* sp., in the rhizosphere of date palms in the Tunisian desert. Symanczik et al. (2014) [56] reported corresponding findings. *Glomus* sp. proved to be the most abundant of the AMF species isolated from all the soil samples examined, proving a significant prevalence. This predominance is due to the unique capacity of *Glomus* species to survive in arid environments, such as the oasis in Figuig, the site of our study.

## 5. Conclusions

This experiment brought to light the existence and variety of AMF within the rhizosphere soil of date palm trees in the Figuig oasis, highlighting the significance of the findings. Soil samples from the ZG and EX sites in the Figuig oasis contained a higher percentage of isolated spores. Additionally, a negative correlation between the soil phosphorus content and the presence of these spores was observed, while a positive association between the soil pH and the presence of these spores was identified. These results confirm the efficacy of mycorrhization in phosphorus-deficient soils. The *Glomus* genus was the most prevalent in the eight studied sites, while the *Sclerocystis* and *Acaulospora* genera were present in extremely low proportions.

Studying the mycorrhizal potential of rhizosphere soils and identifying these fungi, which help plants grow in the dry environment of the Figuig oasis by making them more resistant to different environmental conditions, are proving to be of the utmost importance. These measures pave the way for potential future studies focusing on environmental preservation and sustainable agriculture applications.

## Figures and Tables

**Table 1:** Physicochemical properties of the eight studied soil samples.

Site/Soil Property	ZG	OD	LZ	OS	EH	LB	AR	EX
pH	7.8	7.6	7.4	7.7	7.7	7.6	7.7	8.1
SOM (%)	0.4	0.4	1.8	0.9	1	1.7	0.6	0.4
P_2_O_5_ (ppm)	20.3	37.4	100	78.6	36.3	72	24.1	23.3
K_2_O (ppm)	53	193.5	158.3	176	211	158.3	70.4	105.5
CaCO_3_T (%)	9.5	18.5	22	21	20	21	8	45
Salinity (g/kg)	0.1	0.2	0.7	1.1	0.1	0.9	0.3	1.2
Texture	Clay loam	Clay loam	Clay loam	Clay loam	Clay loam	Clay loam	Sandy clay loam	Sandy clay loam

Aarja (AR); Extension (EX); Elhammam (EH); Laabidate (LB); Lamaiz (LZ); Oudaghir (OD); Ouled slimane (OS); and Zenaga (ZG).

**Table 2:** Number of maize plants showing traces of AMF at successive dilutions in soils sampled from eight sites.

		Repetition ×5					
	Dilution	1	1/4	1/16	1/64	1/256	1/1024
Site							
ZG		5	5	5	5	5	5
OD		5	5	5	5	3	3
LZ		5	5	5	5	3	2
OS		5	5	5	5	5	3
EH		5	5	5	5	4	3
LB		5	5	5	5	3	3
EX		5	5	5	5	5	5
AR		5	5	5	5	4	3

Aarja (AR); Extension (EX); Elhammam (EH); Laabidate (LB); Lamaiz (LZ); Oudaghir (OD); Ouled slimane (OS); and Zenaga (ZG).

**Table 3:** AMF isolated from date palm groves in the oasis of Figuig.

ID	Size	Color	External Wall	Internal Wall	Suspension Hyphae	Abundance	Form	Internal Structures	Wall Number	Single-Spore or Clustered
FIG1	150 µm	Yellow–brown	Present	Present	Present	Mediocre	Globular	Lipid droplets	3	In a cluster
FIG2	120 µm	Brown	Present	Present	Present	Mediocre	Globular	Lipid droplets	2	Single
FIG3	50 µm	Orange	Present	Present	Present	High	Spherical	Lipid droplets	2	Single
FIG4	100 µm	Yellow–brown	Present	Absent	Present	Mediocre	Subglobular	Lipid droplets	2	In a cluster
FIG5	300 µm	Orange yellow	Present	Present	Absent	High	Oval	Lipid droplets	3	In a cluster
FIG6	100 µm	Yellow	Present	Present	Present	High	Subglobular	Lipid droplets	3	In a cluster
FIG7	250 µm	Orange	Present	Absent	Absent	Rare	Globular	Lipid droplets	2	Single
FIG8	130 µm	Red–brown	Present	Present	Present	Mediocre	Globular	Lipid droplets	3	Single
FIG9	100 µm	Brown	Present	Present	Present	High	Oval	Lipid droplets	3	In a cluster
FIG10	120 µm	Light yellow	Present	Present	Absent	Mediocre	Globular	Lipid droplets	2	Single
FIG 11	270 µm	Yellow	Present	Present	Present	Mediocre	Subglobular	Lipid droplets	2	In a cluster

## Data Availability

Not applicable.
